# Expert Consensus on Trendelenburg Position Postless Hip Distraction Technique in Hip Arthroscopy

**DOI:** 10.1111/os.70303

**Published:** 2026-04-21

**Authors:** Yaoting Wang, Lingxing Jiang, Jiakai Sun, Hao Fu, Mingxing Wang, Long Wang, Xiaoqi Kang, Jia Zhang, Jianquan Wang, Yujie Liu, Qingfeng Yin, Chunbao Li

**Affiliations:** ^1^ Department of Sports Medicine, Senior Department of Orthopedics The Fourth Medical Center of Chinese PLA General Hospital Beijing China; ^2^ Department of Orthopedics Peking University Third Hospital (PUTH) Beijing China; ^3^ Department of Orthopedics The Second Qilu Hospital of Shandong University Jinan Shandong China

**Keywords:** complications, expert consensus, hip arthroscopy, post hip distraction, postless hip distraction

## Abstract

Postless hip distraction technique is a novel traction method for hip arthroscopy developed in recent years. Studies have shown that, compared with the traditional post hip distraction technique, it can effectively solve perineal complications, lower the technical threshold of hip arthroscopy, and improve patients' postoperative satisfaction. The standardization of this technique is expected to promote the advancement and popularization of hip arthroscopy. To this end, this Consensus identified 17 questions of the greatest clinical concern that were selected through systematic literature retrieval and evidence quality evaluation, and in combination with clinical practice, using the Grading of Recommendations Assessment, Development and Evaluation (GRADE) system and the Reporting Items for Practice Guidelines in Healthcare (RIGHT), and 20 evidence‐based medical recommendations were finally formulated, aiming to improve the standardization and scientific nature of the postless hip distraction technique and provide a basis for improving the quality of patient‐centered medical services.

## Graphical abstract

This work evaluates the Trendelenburg position postless hip distraction technique for hip arthroscopy, highlighting its safety, efficacy, cost‐effectiveness, and key clinical application points.

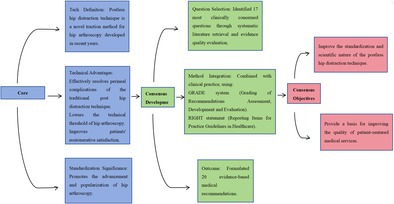

## 1 Background of Consensus Development

With the global population aging and the promotion of national fitness campaign, the incidence of hip diseases has increased significantly. Hip arthroscopy is an effective method for treating hip diseases, which can prevent the hip joint from developing into end‐stage arthritis. In recent years, the number of hip arthroscopic surgeries has shown an exponential upward trend [1, 2]. Hip arthroscopic surgery requires traction of the lower limb to distract the hip joint space for surgical manipulation in the central compartment. Most surgeons perform this procedure with the patient in the supine position, using a perineal post to counteract sustained traction on the lower limb [3]. Due to the relatively complexity of hip arthroscopic surgery, surgeons face a steep learning curve and long operation duration. Intraoperative use of the perineal post for auxiliary traction can cause prolonged perineal compression, potentially leading to soft tissue injuries such as scrotal or labial necrosis and vaginal tearing, or nerve injury complications such as perineal pain, hypaesthesia, and erectile and ejaculatory dysfunction [4, 5]. Even surgeons with high surgical volumes and proficient techniques cannot avoid the occurrence of perineal complications. Nwachukwu et al. conducted a study in 218 adolescents undergoing hip arthroscopy and reported an overall complication rate of 1.8%, with perineal nerve palsy accounting for 50% of these complications [6]. A systematic review showed that in 17 prospective studies using perineal post‐assisted traction, the incidence of perineal complications was 7.1%, which is much higher than the 1.4% incidence reported in 74 retrospective studies [7, 8]. Therefore, how to effectively reduce complications caused by post hip distraction is a clinical challenge.

In 2007, scholars first proposed a postless hip distraction technique. By eliminating the perineal post while ensuring the smooth progress of the surgery, this technique effectively resolves soft tissue and nerve‐related complications caused by perineal compression [9]. Studies have indicated that compared to the traditional post hip distraction technique, the postless hip distraction technique effectively resolves perineal complications, reduces the technical threshold for hip arthroscopy, and improves patients' postoperative satisfaction. A study demonstrated that surgical duration and postoperative pain levels were similar between postless and post hip traction techniques, but the postoperative hospital stay was shorter in the postless hip traction group [10]. Another study indicated that compared to traditional post hip distraction, hip arthroscopy using postless hip traction did not cause significant reduction in venous blood flow or neurological changes in the affected limb. Muscle tissue injury was subclinical and transient, and no cases of perineal injury were observed during the study [11]. In recent years, the postless hip traction technique has gained increasing recognition and application among hip arthroscopists. However, various postless hip traction techniques have been reported domestically and internationally, including the Trendelenburg position method, Tutankhamun technique, and pink pad technique. Different methods have their own advantages and disadvantages in terms of operational convenience, applicability across patient populations, and traction stability, with no unified standard established. Currently, there are no guidelines and consensus documents on the postless hip distraction technique either domestically or internationally, which limits the promotion and popularization of this technique. Therefore, there is an urgent need for an expert consensus on the postless hip distraction technique in hip arthroscopy to better guide intraoperative manipulation and promote the technique.

## 2 Formulation Methods of the Consensus

**FIGURE 1:**
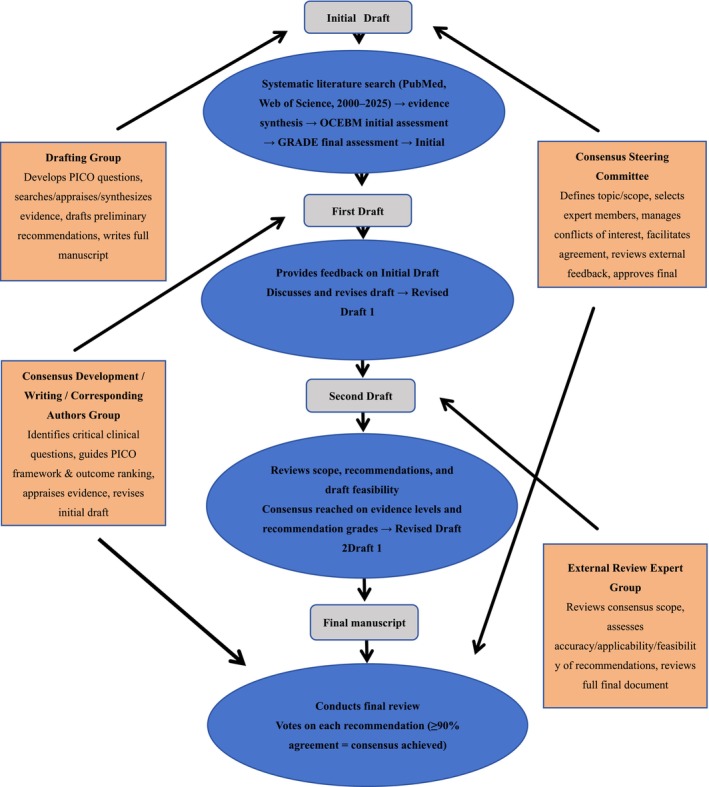
Flowchart of the consensus development process.

The consensus invited a total of 40 experts covering sports medicine, anesthesiology, and nursing (see Appendix S1 for further details). The consensus development process is shown in Figure 1. The Expert Committee for the Development of The Consensus was composed of a Consensus Steering Committee, a Consensus Development Expert Group, a Writing Expert Group, an External Review Expert Group, a Corresponding Authors Group, and a Drafting Group. The Consensus Steering Committee was responsible for: (1) defining the consensus topic and scope; (2) selecting members for all expert Groups; (3) managing conflicts of interest; (4) facilitating agreement on recommendation statements; (5) reviewing feedback from the external review Group; (6) approving the final consensus document; and (7) overseeing the entire consensus development process. The Consensus Development Expert Group, Writing Expert Group, and Corresponding Authors Group were responsible for: (1) identifying critical clinical questions requiring resolution; (2) guiding the Drafting Group in formulating the PICO (Population, Intervention, Control, Outcome) framework and ranking outcome measures; (3) appraising and interpreting evidence‐based medical literature; and (4) revising the initial consensus draft for review by the Steering Committee. The External Review Group was responsible for: (1) reviewing the scope of the consensus; (2) assessing the accuracy, applicability, and feasibility of the recommendation statements; and (3) reviewing the full consensus document after the recommendations were finalized. The Drafting Group was responsible for: (1) developing specific clinical PICO questions; (2) searching for, appraising, and synthesizing the body of evidence; (3) drafting preliminary recommendation statements; and (4) writing the full consensus document.

The consensus was developed using a clinical question‐oriented approach and a modified Delphi method, which involved two rounds of online questionnaire surveys and one round of online discussion. The specific process was as follows: (1) The expert panel systematically searched relevant literature from databases including PubMed, Web of Science from 2000 to 2025. English search terms included “Hip arthroscopy”, “Postless hip distraction”, “Post hip distraction”, “Complications”, “Expert Consensus”, and so forth. English literature including systematic reviews, meta‐analyses, randomized controlled trials, and cohort studies were included. On the basis of reviewing, summarizing, and sorting out the literature, the drafting group prepared the initial draft of the consensus. The evidence level and recommendations were initially assessed according to the 2011 Oxford Centre for Evidence‐Based Medicine (OCEBM) criteria (Tables 1 and 2). The Grading of Recommendations Assessment, Development and Evaluation (GRADE) system was then used for the final assessment of evidence quality and strength of recommendations. Evidence quality was classified into four levels: high, moderate, low, and very low; recommendations were classified into two grades: strong recommendation and weak recommendation (see Appendix S2 for further details) (2) The Consensus Development Expert Group provided feedback on the initial draft, which was then discussed and revised by the Writing Group (3) The External Review Group further reviewed the revised draft, leading to a consensus on the levels of evidence and recommendation grades (4) The finalized draft was submitted to the Steering Committee for final input. Ultimately, all members of the Consensus Development Expert Group voted on each recommendation. A consensus was achieved when the agreement rate reached 90% or higher. This consensus addresses 17 clinical questions that are of high relevance in clinical practice yet lack standardized approaches. It aims to provide practical guidance and reference for clinicians and therapists involved in related fields.

**TABLE 1:** Oxford Centre for Evidence‐Based Medicine levels of evidence (2011 version).

Level of evidence	Description
1	Systematic reviews of RCTs, all‐or‐none studies, or observational studies with large effect sizes
2	Individual RCTs, or observational studies with large effect sizes
3	Non‐randomized controlled cohort/follow‐up studies
4	Case series, case–control studies, or historically controlled studies
5	Mechanism‐based reasoning

*Note:* Grading may be downgraded based on factors such as study quality, imprecision, indirectness (mismatch between the PICO of the cited studies and the PICO addressed by the consensus recommendation), and small effect size; it may be upgraded based on a large effect size. PICO stands for Population, Intervention, Comparison, Outcome; RCT stands for Randomized Controlled Trial.

**TABLE 2:** Oxford Centre for Evidence‐Based Medicine grades of recommendation (2011 version).

Grade of recommendation	Description
A	Consistent level 1 studies
B	Consistent level 2 or 3 studies; or extrapolations from level 1 studies
C	Consistent level 4 studies; or extrapolations from level 2 or 3 studies
D	Level 4 studies; or any level of evidence that is inconsistent or uncertain

*Note:* The strength of recommendations follows the principles of the 2011 Oxford grading system; however, in the formulation of certain recommendations, the final grade was determined based on specific considerations in clinical practice.

## 3 Main Content of the Consensus

### 3.1 Definition of Postless Hip Distraction Technique in Hip Arthroscopy

#### 3.1.1 Clinical Question 1: What Is the Postless Hip Distraction Technique for Hip Arthroscopy?

*Recommendation* 1: The postless hip traction technique in hip arthroscopy refers to a traction method that achieves sufficient distraction of the affected hip joint by adjusting the patient's surgical position and/or utilizing the friction generated between the fixed trunk and the operating table to counteract the traction force applied to the affected limb toward the foot end. This concept encompasses various traction methods such as the Trendelenburg position and pink pad technique. These methods are expected to be applicable to a variety of traditional hip surgeries requiring lower limb traction, including femoral neck fractures and intertrochanteric fractures (Strong recommendation, evidence level C).

*Summary of evidence*: The traditional post hip distraction technique is prone to cause complications such as nerve injury and soft tissue injury in the perineal region. Nwachukwu et al. investigated 218 adolescents undergoing hip arthroscopy and reported an overall complication rate of 1.8%, with perineal nerve palsy accounting for 50% of these complications [6]. A systematic review showed that in 17 prospective studies using perineal post‐assisted traction, the incidence of perineal complications was 7.1%, which is much higher than the 1.4% incidence reported in 74 retrospective studies [7, 8]. Therefore, how to effectively reduce the complications caused by post hip distraction is a clinical challenge.

Some scholars have proposed the postless traction technique. Its principle is to counteract the traction force on the lower limb by fixing the trunk, utilizing the patient's own body weight and the friction between the body and the operating table, fundamentally eliminating perineal compression caused by the perineal post. Currently reported postless hip traction techniques in the literature include: Trendelenburg position method, beanbag technique, Tutankhamun technique, yoga mat technique, and pink pad technique. Studies have shown that the clinical efficacy of these techniques can meet surgical requirements and that they can effectively reduce the occurrence of perineal complications [12, 13, 14, 15]. In addition, a study by Aprato et al. has proved that the postless hip distraction technique also achieved satisfactory results for intraoperative traction of lower limb fractures such as hip fractures and femoral shaft fractures [16].

#### 3.1.2 Clinical Question 2: What Are the Advantages and Disadvantages of Different Postless Hip Distraction Techniques?

*Recommendation* 2: The postless hip distraction technique covers various implementation methods such as the Trendelenburg position method, pink pad technique, and yoga mat technique. Among them, the Trendelenburg position postless hip distraction technique offers comprehensive advantages, including low equipment requirements, convenient operation, flexible intraoperative position adjustment, and easy popularization. Other methods have disadvantages such as cumbersome operation, potential interference with anesthesia, high equipment costs, and limited population adaptability. Overall, the Trendelenburg position postless hip distraction technique demonstrates leading performance in terms of cost‐effectiveness, practicality, safety, and population adaptability, and should be regarded as the preferred method for postless hip distraction in hip arthroscopy (Strong recommendation, evidence level D).

*Summary of evidence*: Currently reported postless hip distraction techniques include the beanbag technique [9], Tutankhamun technique [12], yoga mat technique [15], pink pad technique [13, 17], HAPPI technique [18], Trendelenburg position postless hip distraction technique [19], postless hip distraction technique using a hip distractor [18], and the Postless distraction technique with no additional equipment [20]. Based on different traction principles, current postless hip distraction techniques are mainly divided into three categories, including: ① Counteracting traction by binding and fixing the trunk and upper limbs; ② Counteracting traction by increasing the friction between the body and the table surface; and ③ Counteracting traction by adjusting the position and utilizing the patient's own gravity. All the aforementioned techniques can eliminate perineal complications and achieve adequate hip joint distraction for surgical needs. However, they still present certain drawbacks.

The beanbag technique [9] and the Tutankhamun technique [12] mainly rely on the fixation of the trunk and upper limbs to counter traction, resulting in a relatively single counter‐traction force. The beanbag technique still uses the perineal post during initial positioning and general anesthesia induction. Therefore, this technique only reduces the duration of perineal post use, rather than completely eliminating its use. The Tutankhamun technique is complicated to operate. Excessive restraint of the upper limbs and chest during surgery may affect anesthesia and make intravenous fluid infusion via upper limb veins not possible. The postless hip distraction technique using a hip distractor [18], the pink pad technique [21], the yoga mat technique [15], and the HAPPI technique [22] all require additional accessories or specialized equipment, which increases the cost and makes the operation more complicated. Furthermore, the use of a hip distractor [18] is an invasive procedure. The use of disposable traction pins increases the cost and operation duration, and brings risks such as iatrogenic fractures, infections, and increased postoperative pain. For patients with a low body weight, the Trendelenburg position postless hip distraction technique may require increasing the Trendelenburg angle to maintain sufficient traction, which brings new challenges to surgical manipulation and fluoroscopy. Specialized perineal post‐free traction tables require purchasing dedicated equipment, which is expensive and also involves an additional learning curve [23].

Clinically, it is recommended to select an appropriate postless hip distraction technique based on local conditions, prioritizing techniques that are minimally invasive, do not require additional equipment or accessories, and do not require maintaining the Trendelenburg position continuously.

#### 3.1.3 Clinical Question 3: What Is Trendelenburg Position Postless Hip Distraction Technique for Hip Arthroscopy?

*Recommendation* 3: The Trendelenburg position postless hip distraction technique for hip arthroscopy refers to a traction method that achieves sufficient distraction of the hip joint to meet surgical operational requirements by adjusting the operating table to a Trendelenburg position with an angle of 10°–15° without placing a perineal post as a counter‐traction fulcrum, and using restraint straps to bind and fix the trunk at the thoracoabdominal junction and the contralateral inguinal region, relying on the patient's gravity and the friction between the body and the operating table to counteract lower limb traction force (Strong recommendation, evidence level D).

*Summary of evidence*: The postless hip distraction technique with Trendelenburg position in hip arthroscopy referred to in these recommendations is essentially a specific application of the Trendelenburg position in hip arthroscopy traction. Its core concept is not relying on a perineal post as the countertraction fulcrum. Instead, the patient's own body weight in the 10°–15° Trendelenburg position serves as the primary counterforce, assisted by torso fixation and bed friction to achieve hip joint distraction. As an important type of postless hip distraction technique, its key feature is replacing the counteraction of the perineal post by adjusting patient positioning. This fundamentally eliminates perineal compression caused by the perineal post, which distinguishes it from conventional traction and other postless hip distraction methods. Relevant studies have confirmed that this technique can significantly reduce the incidence of perineal complications [12, 13, 14, 15].

### 3.2 Rationale for Trendelenburg Position Postless Hip Distraction Technique in Hip Arthroscopy

#### 3.2.1 Clinical Question 4: What Is the Theoretical Basis for the Trendelenburg Position Postless Hip Distraction Technique in Hip Arthroscopy?

*Recommendation* 4: When the Trendelenburg position is adopted, the patient's own body weight generates a component force toward the head end under the inclination angle of 10°–15° of the operating table. This component force is superimposed with the maximum static friction between the body and the operating table to jointly form a counterforce against the physical traction force applied to the affected limb toward the foot end. When this counterforce is greater than the traction force, effective distraction of the hip joint can be achieved (Strong recommendation, evidence level C).

#### 3.2.2 Clinical Question 5: Can the Trendelenburg Position Postless Hip Distraction Technique in Hip Arthroscopy Sufficiently Distract the Hip Joint Space?

*Recommendation* 5: The Trendelenburg position postless hip distraction technique can sufficiently distract the hip joint space, achieving a distraction distance of 8–10 mm, which meets the operational requirements of hip arthroscopy. There is no statistical difference in the traction effect compared with the traditional post hip distraction technique. The distraction effect in patients who are overweight or have joint stiffness should be judged based on clinical actual conditions (Strong recommendation, evidence level C).

*Summary of evidence*: Adjusting the operating table to the Trendelenburg position generates a component force of the patient's own body weight downward along the table surface. Since the Trendelenburg angle is a variable, it can be adjusted as needed. The larger the angle, the greater the gravitational component force.

There is a natural friction between the human body and the table surface, which depends on the patient's own body weight and the friction coefficient between the skin and the operating table. Both gravity and the friction coefficient are non‐variable. We increase the downward force by fixing the trunk, thereby increasing the friction [24].

The combination of gravitational component force and the friction force can counteract the traction force applied to the affected limb toward the foot end (to achieve 8–10 mm distraction of the hip joint).

Based on the above theory, achieving hip joint distraction using the postless hip distraction technique is more challenging in patients with lower body weight. Conversely, the postless hip distraction technique is more conducive to hip joint distraction in patients with higher body weight.

### 3.3 Advantages of the Trendelenburg Position Postless Hip Distraction Technique Over the Traditional Post Hip Distraction Technique in Hip Arthroscopy

#### 3.3.1 Clinical Question 6: Can the Trendelenburg Position Postless Hip Distraction Technique in Hip Arthroscopy Effectively Resolve Perineal Complications Caused by the Traditional Traction Techniques?

*Recommendation* 6: Since the Trendelenburg position postless hip distraction technique no longer uses a perineal post, it fundamentally eliminates the continuous compression of perineal soft tissues by the traditional perineal post, significantly reducing or avoiding the occurrence of perineal complications (Strong recommendation, evidence level C).

*Summary of evidence*: Previous studies have shown that with the post hip distraction technique, a lower limb traction force of 200 N is sufficient to distract the hip joint space by 8–10 mm, which meets the operational requirements for the central compartment of hip joint [13]. Multiple studies on various postless hip distraction techniques reported in the literature have proven that such techniques can all achieve adequate hip joint distraction for arthroscopic procedures. In terms of surgical efficacy, there is no statistical difference between the postless hip distraction technique and the traditional post hip distraction technique [12, 13, 14, 15].

It has been reported in the literature that the traction force required for the postless hip distraction technique is less than that for the traditional post hip distraction technique. A study by Parkes in 2023 included a total of 195 patients, of whom 94 were in the postless hip distraction group and 101 in the post hip distraction group. The maximum traction force (70.83 ± 14.87 kg vs. 96.31 ± 16.53 kg; *p* < 0.001) and the maintained traction force (55.89 ± 14.91 kg vs. 68.31 ± 14.77 kg; *p* < 0.001) in the postless hip distraction group were both lower than those in the post hip distraction group [25].

#### 3.3.2 Clinical Question 7: What Is the Difference in the Traction Force Required Between the Trendelenburg Position Postless Hip Distraction Technique and the Traditional Post Hip Distraction Technique in Hip Arthroscopy?

*Recommendation* 7: Compared with the traditional post hip distraction technique, the traction force applied to the distal end of the affected limb is slightly smaller when achieving the ideal hip joint distraction space using the Trendelenburg position postless hip distraction technique (Weak recommendation, evidence level C).

*Summary of evidence*: A controlled study by O'Neill et al. included a total of 105 patients, of whom 51 patients underwent the postless hip distraction. The continuous traction force (67.5 ± 14.0 kgf vs. 55.8 ± 15 kgf) and the maximum traction force (96.0 ± 16.6 kgf vs. 69.9 ± 14.1 kg) in the postless hip distraction group were significantly lower than those in the post hip distraction group. Similar studies have also confirmed that the traction force required for the postless hip distraction technique is smaller than that for the traditional post hip distraction technique [25].

#### 3.3.3 Clinical Question 8: What Other Significant Advantages Does the Trendelenburg Position Postless Hip Distraction Technique in Hip Arthroscopy Have Over the Traditional Post Hip Distraction Technique?

*Recommendation* 8: The Trendelenburg position postless hip distraction technique effectively relieves the compression of perineal soft tissues, relaxing the traction time limit for hip arthroscopy to a certain extent (maximum 2 h). However, the risk of sciatic nerve and lower limb nerve stimulation caused by excessive traction still needs to be considered (Strong recommendation, evidence level C).

*Recommendation* 9: The Trendelenburg position postless hip distraction technique facilitates dynamic intraoperative observation and tests of hip flexion, internal rotation, and external rotation (Weak recommendation, evidence level: D).

*Summary of evidence*: Studies have proven that the traction force required for the postless hip distraction technique is smaller than that for the traditional post hip distraction technique [25, 26]. Simultaneously, by eliminating perineal compression, the intraoperative traction duration is not strictly limited to 2 h, theoretically allowing for consecutive bilateral surgery.

Techniques such as the yoga mat method, pink pad technique, and plastic sheet fixation method all use additional materials, resulting in a higher cost. The postless distraction technique with no additional equipment only uses routine materials in the operating room, does not require special equipment or consumables, is simple to prepare, and does not incur extra costs [12, 13, 14, 15].

Analysis based on principles shows that different postless hip distraction techniques do not require specialized traction tables and can achieve distraction using ordinary orthopedic traction tables.

Due to trunk and upper limb binding and fixation, the Tutankhamun technique and plastic sheet fixation method may interfere with anesthesia due to excessive restraint of the upper limbs and chest during surgery and make intravenous fluid infusion via upper limb veins not possible. Other postless hip distraction techniques do not involve binding and compression of the trunk, thus having no significant impact on anesthesia and respiration. In addition, studies have proven that maintaining the Trendelenburg position at an angle of less than 15° has no significant impact on respiration, anesthesia, and vital signs during surgery [12, 13, 14, 15].

For patients with femoroacetabular impingement (FAI), after cam lesion resection and shaping, the absence of a perineal post obstruction allows for dynamic assessment of the shaping adequacy under arthroscopic monitoring with the hip in flexion, internal rotation, and external rotation [20].

Studies have reported that compared with the traditional post hip distraction technique, the postless hip distraction technique is associated with faster postoperative recovery and shorter hospital stay [25, 26].

#### 3.3.4 Clinical Question 9: Does the Trendelenburg Position Postless Hip Distraction Technique in Hip Arthroscopy Have Cost‐Effectiveness Advantages Over the Traditional Post Hip Distraction Technique?

*Recommendation* 10: The Trendelenburg position postless hip distraction technique significantly reduces the medical behaviors, medical costs, and hospital stay length due to post‐operative perineal complications, thus having certain cost‐effectiveness advantages (Weak recommendation, evidence level C).

*Summary of evidence*: The postless hip distraction technique does not significantly increase the direct costs of surgery, drugs, examinations, etc., nor does it require purchasing additional equipment and instruments [20]. Compared with the traditional post hip distraction technique, the postless hip distraction technique does not reduce the blood flow in the lower limb or damage the nerve function of the affected limb, and can significantly reduce complications such as perineal and inguinal numbness, pain, and swelling [11, 22], thereby effectively reducing corresponding costs of treatment, rehabilitation, work loss, etc., and shortening hospital stay duration.

### 3.4 Key Points of the Trendelenburg Position Postless Hip Distraction Technique in Hip Arthroscopy

#### 3.4.1 Clinical Question 10: What Are the Indications and Contraindications of the Trendelenburg Position Postless Hip Distraction Technique in Hip Arthroscopy?

*Recommendation* 11: The Trendelenburg position postless hip distraction technique is suitable for assisting in the performance of surgeries for diseases indicated for routine hip arthroscopy, including but not limited to femoroacetabular impingement syndrome (FAIS), borderline developmental dysplasia of the hip (BDDH), synovial chondromatosis of the hip joint, pigmented villonodular synovitis, gout, hip labral tears, loose bodies, foreign bodies, purulent arthritis, and femoral head necrosis. This technique has no special restrictions on the patient's gender, age, and body mass index (BMI) (Strong recommendation, evidence level C).

*Summary of evidence*: With the in‐depth study on hip arthroscopy and related diseases, the surgical indications are still expanding. Wu Yidong et al. [27] summarized the surgical indications for hip arthroscopy, including femoroacetabular impingement syndrome (FAIS), hip labral tears, ligamentum teres injuries, hip cartilage injuries, posterior hip dislocation, femoral head fractures, inflammatory hip diseases such as ankylosing spondylitis and rheumatoid arthritis, intra‐articular hip tumors and periacetabular cysts, femoral head necrosis, and congenital developmental hip diseases such as developmental hip dysplasia and borderline developmental hip dysplasia. In a prospective study by Kraeutler et al. [20] using the postless hip distraction technique, the diseases that can be treated or operations that can be performed using this technique include: treatment, debridement, repair, and reconstruction of labral tears; treatment of femoral osteoplasty, acetabular osteoplasty (FAI); treatment of femoral head necrosis; iliotibial band lengthening, repair of hip cartilage injuries, greater trochanteric bursectomy, adductor lengthening, and joint capsule reconstruction. Summarizing similar studies, it is found that the postless hip distraction technique is feasible for hip arthroscopy in cases of femoroacetabular impingement syndrome (FAIS), hip labral tears, ligamentum teres injuries, hip cartilage injuries, posterior hip dislocations, femoral head fractures, inflammatory hip diseases such as ankylosing spondylitis and rheumatoid arthritis, intra‐articular hip tumors and periacetabular cysts, femoral head necrosis, and congenital developmental hip diseases such as developmental hip dysplasia and borderline developmental hip dysplasia. In addition, some studies have proven that the postless hip distraction technique also achieved satisfactory results for intraoperative traction of lower limb fractures such as hip fractures and femoral shaft fractures [17, 28]. Aprato et al. [17] reported the application of the postless hip distraction technique in the reduction and intramedullary nailing of femoral shaft fractures. Since the patient had a concomitant pelvic fracture and perineal skin and soft tissue injuries, they chose to use the postless hip distraction technique to avoid pelvic fracture displacement and aggravation of the pre‐existing perineal injury.

*Recommendation* 12: Contraindications for the Trendelenburg position postless hip distraction technique primarily relate to the potential impact of the Trendelenburg position on the patient, specifically including: patients with cardiopulmonary insufficiency, patients with elevated intracranial or intraocular pressure, patients with severe gastroesophageal reflux and a full stomach, patients with cerebrovascular diseases, and patients with superior vena cava diseases.

Relative contraindications for this technique include patients with deep acetabulum or excessive acetabular coverage, patients with moderate‐to‐severe hip osteoarthritis (OA), patients with joint adhesions or stiffness, and patients with a history of lower limb neuropathy or injury (Weak recommendation, evidence level: C).

*Summary of evidence*: Wu Yidong et al. [27] believe that open hip trauma and active systemic infections are absolute contraindications for hip arthroscopy. Hip arthroscopy is not recommended for patients with severe hip osteoarthritis because the probability of undergoing total hip replacement in the short term after surgery is extremely high. Hip ankylosis, fibrosis, and joint capsule contracture may lead to a narrow arthroscopic field of view, making surgical manipulation difficult and affecting the postoperative outcomes.

A study [19] found that the most important variables determining the initial traction force in patients undergoing postless hip distraction were gender (*p* < 0.001), body weight (*p* < 0.001), and lateral center‐edge angle (*p* < 0.01) in sequence. Patients with joint laxity, female patients, those with a higher BMI (> 30 kg/m^2^), and those with developmental hip dysplasia are easier to distract, while elderly males with hip OA and joint stiffness, young lean males, and those with large cam lesions are more difficult to distract. Wendy M Meek et al. [29] reported a 27‐year‐old male patient scheduled for hip arthroscopy with postless hip distraction who failed the postless hip distraction due to the deep acetabulum and subsequently underwent arthroscopic labral repair using perineal post traction. Larson [21] believed that the risk factors for failed traction with the postless hip distraction technique include young and stiff male patients and severe excessive acetabular coverage; if insufficient traction occurs, using a perineal post for traction is also safe and feasible.

#### 3.4.2 Clinical Question 11: What Are the Potential Adverse Events of the Trendelenburg Position Postless Hip Distraction Technique in Hip Arthroscopy?

*Recommendation* 13: The adoption of the Trendelenburg position postless hip distraction technique may lead to risks of significant patient displacement and falling off the table due to excessive traction force; local soft tissue and nerve injuries may occur if restraint straps are fixed too tightly; it may cause increased intrathoracic pressure, intracranial pressure, and intraocular pressure; and postoperative pain or discomfort in the lower back, pelvic region, knee, and ankle and foot regions may occur. Intraoperative protection should be strengthened to reduce related risks (Weak recommendation, evidence level: C).

*Summary of evidence*: The postless hip distraction technique can achieve sufficient hip joint distraction in most cases, with the distraction gap ranging from 11 to 20 mm. The incidence of insufficient hip joint distraction is no more than 1% of the total cases, which mainly occurs in the early stage of using this technique when it is not yet proficiently applied and when preoperative hip radiograph review is inadequate and surgical preparation is insufficient [19]. Meek et al. [29] reported a patient who failed postless hip distraction due to a deep acetabulum and the procedure was terminated, and then switched to post hip distraction for arthroscopic labral repair. Another study on the postless hip distraction technique included 1000 patients undergoing hip arthroscopy with this technique, among whom two patients experienced insufficient traction in a total of four hip arthroscopic surgeries, all of which were within the first 100 surgeries performed by the operator using the postless hip distraction technique [19]. For cases of unsuccessful postless hip distraction, it is recommended to switch to post hip distraction midway during surgery. Robert Kollmorgen et al. commented on the postless hip distraction technique and said that “Regardless of post‐free distraction technique, there have been no reported patient safety nor groin complications” [30].

Although there are no reports of patients falling off the table in the existing literature, the risk exists, and attention should be paid by surgeons, anesthesiologists, and assistants during traction.

The postless hip distraction technique may cause sub‐clinical injury to the lower limb blood vessels, posing a risk of deep vein thrombosis (DVT). A prospective study by Welton KL et al. [11] found that the postless hip distraction technique resulted in a significant reduction in the blood flow in the common femoral and popliteal veins of both lower limbs, but the reduction ratio did not exceed 50%, and there was no significant difference between the surgical and non‐surgical sides. Although the positive rate of D‐dimer was increased significantly 7–12 days after surgery (55.7%, 95% CI 33.0%–74.0%, *p* < 0.01), there was no significant correlation between the elevated D‐dimer level and the decreased venous blood flow, and no patient was clinically diagnosed with DVT. However, in another report of over 1000 surgeries using the postless hip technique, two cases developed DVT, which were related to the patient's own risk factors for DVT (BMI 35 kg/m^2^, complicated with diabetes, and older age) [19].

In terms of the impact on lower limb muscle tissue, studies have shown that 22.5% of patients using the postless hip distraction technique experienced an immediate and significant increase in postoperative creatine kinase (CK) (95% CI, 9.0%–46.0%, *p* = 0.04). At 7–12 days after surgery, CK levels tended to remain elevated compared with baseline, and 20.5% of patients had levels higher than the upper limit of normal (95% CI, 8.0%–42.6%, *p* = 0.05). Secondary analysis showed that patients undergoing consecutive bilateral hip arthroscopy were more likely to have elevated CK levels immediately after surgery (odds ratio, 22.5; *p* = 0.02). Despite the significant changes in CK levels, no clinical manifestations were observed [11].

The post hip distraction technique may cause urological dysfunction and nerve injuries, with the overall postoperative numbness rate ranging from 12% to 30.9% [25, 31]. Postoperative nerve injury after hip arthroscopy is related to traction duration and traction force. Telleria et al. reported that for every 1‐pound increase in traction force, the probability of nerve events increases by 4% [32]. A prospective study comparing postoperative inguinal‐related complications after hip arthroscopy with and without perineal post traction [31] found that no inguinal complications or sexual dysfunction occurred after postless hip distraction, but one case (3%) and three cases (12%) developed urinary dysfunction and foot numbness, respectively. Another similar prospective study [25] found that among 94 patients (mean age: 30.4 years) using the postless hip distraction technique, the overall postoperative numbness rate was 30.9% (29/94), including inguinal numbness in one case (1.1%), foot numbness in six cases (6.4%), lateral thigh numbness in 20 cases (21.3%), and nerve root radiation injury in four cases (4.3%). The overall incidence of numbness and the incidence of inguinal numbness were significantly lower than those in the post hip distraction group, and there was no statistically significant difference in other lower limb nerve injuries. They believed that lateral thigh numbness was related to the injury of the lateral femoral cutaneous nerve during incision creation.

#### 3.4.3 Clinical Question 12: How to Position the Patient for the Trendelenburg Position Postless Hip Distraction Technique in Hip Arthroscopy?

*Recommendation* 14: The Trendelenburg angle generally ranges from 10° to 15°. For male patients, low‐weight patients, and patients with high muscle mass, the inclination angle may need to be appropriately increased (Strong recommendation, evidence level C).

*Summary of evidence*: Hip arthroscopy requires traction of the affected lower limb to facilitate surgical manipulation. The Trendelenburg position can counteract the lower limb traction force by utilizing body gravity and the friction between the body and the operating table [11]. Many scholars at home and abroad have conducted research on perineal post‐free positions. Wang et al. [20] reported that a Trendelenburg angle of BMI can meet the requirements of postless hip distraction; generally speaking, 10° is sufficient to achieve a satisfactory traction effect. Kraeutler et al. [33] performed perineal post‐free hip arthroscopy on 87 patients and concluded that a Trendelenburg angle of 3°–15° can achieve lower limb traction. The surgical position angle reported by Kollmorgen et al. [14] was 0°–15°.

Factors such as age, gender, and body weight affect the selection of the position angle. Younger patients, male patients, or patients with high muscle mass typically require greater traction force, necessitating adjustment of the inclination angle [34]. Lower body weight also correlates with the need for a greater inclination angle [35]. Even for patients with a lower body weight, an inclination angle of no more than 25° is sufficient to achieve traction effect. The consensus recommends adopting a Trendelenburg angle of 0°–15° for postless hip distraction according to individual patient differences.

*Recommendation* 15: During surgery, the traction can be released and the operating table can be adjusted to the horizontal position after the central compartment of the hip joint has been treated (Strong recommendation, evidence level C).

*Summary of evidence*: Since the Trendelenburg position carries risks such as increased cardiopulmonary load, intracranial pressure, intraocular pressure, and degree of gastroesophageal reflux [36], which is particularly obvious in obese patients, the duration of this position should not be too long. After the central compartment of the hip joint has been treated, traction should be removed, the patient should be adjusted to the supine position, and subsequent operations should be continued; repeated traction of the lower limb is not recommended.

#### 3.4.4 Clinical Question 13: How to Fix the Trunk and Limbs for the Trendelenburg Position Postless Traction Technique in the Hip Arthroscopy?

*Recommendation* 16: The patient's trunk should be in contact with the sheets on the operating table, and the buttocks should be placed 10 cm away from the end of the operating table. Upper limbs are routinely fixed in a manner that does not interfere with surgical manipulation. Restraint straps are applied at the patient's thoracoabdominal junction and the contralateral inguinal region to fix the trunk. Attention should be paid to avoid fixing the restraint straps too tightly, with a space of one finger between the straps and the trunk to avoid compromising respiration and blood circulation (Strong recommendation, evidence level C).

*Summary of evidence*: Limb fixation is crucial for intraoperative fluoroscopy and arthroscopic manipulation. Multiple studies [18, 20] have pointed out that applying counter‐traction using a restraint strap in the contralateral inguinal region is necessary to stabilize the patient's body. Pressure sores are prone to occur at the restraint strap sites and bony prominences such as the feet, which require protection with soft pads [37, 38]. Due to the special anatomical structure of the femoral neck with an anteversion angle of 15°–20°, Mei‐Dan et al. [29] recommend 15° internal rotation of the affected hip to make the femoral neck contour clearer. There is currently no unified opinion on the fixation of the trunk. Salas et al. [12] adopted a Tutankhamun position, with the patient's upper limbs crossed on the chest and the trunk fixed with adhesive tape. However, this method is inconvenient for observing the fluid infusion and respiration. Wang et al. [35] used thickened restraint straps to fix the trunk at the thoracoabdominal junction, with the affected upper limb retracted for protection and the contralateral upper limb abducted to facilitate observation of fluid infusion.

#### 3.4.5 Clinical Question 14: What Anesthesia Method Is Recommended for the Trendelenburg Position Postless Hip Distraction Technique in Hip Arthroscopy?

*Recommendation* 17: The Trendelenburg position is one of the commonly used standard positions in clinical practice, with an angle range generally of 10°–30°, which can be adjusted according to surgical needs. Using a larger Trendelenburg angle after anesthesia induction usually affects the patient's intracranial pressure, intraocular pressure, and cardiopulmonary function. In hip arthroscopy, maintaining the Trendelenburg angle within 10°–15° has no significant impact on respiration and anesthesia. However, it is recommended to slowly adjust the angle when positioning the patient and closely observe the patient's response. In addition, this position may lead to an increase in the epidural anesthesia plane, so general anesthesia is recommended as the first choice. Moreover, general anesthesia is more convenient for flexibly adjusting the dose of muscle relaxants during surgery, ensuring the smooth progress of the operation (Weak recommendation, evidence level C).

*Summary of evidence*: Previous studies have shown that the postless hip distraction technique can be performed in various ways, and the patient's position is mostly supine and/or adjusted to a Trendelenburg angle of BMI. Studies on the risks of the Trendelenburg position have reported that although a higher BMI facilitates hip distraction, patients with a higher BMI in the Trendelenburg position are prone to an overall increase in airway resistance and the risk of atelectasis. In a study of gynecological robot‐assisted laparoscopic surgery, six patients (accounting for 14% of the patients who needed to switch surgical methods and 0.6% of all patients) could not tolerate the Trendelenburg position due to difficulty in achieving sufficient tidal volume and low blood oxygen saturation (SpO_2_) [39]. Maintaining the Trendelenburg position for 30 min is associated with cerebral hyperperfusion and reduced cerebral oxygen extraction, but does not cause significant brain tissue metabolic disorders [23]. There is limited literature on postoperative cognitive complications in patients without intracranial lesions undergoing surgery in a steep Trendelenburg position, and there is insufficient evidence to indicate that short‐ or long‐term neurological complications will occur after surgery in the Trendelenburg position [40]. The Trendelenburg position can increase intraocular pressure (IOP) and affect patients' visual acuity. However, the preventive use of dorzolamide‐timolol drops after anesthesia induction can significantly reduce IOP; for patients with IOP greater than 40 mmHg during surgery, administration of dorzolamide‐timolol can prevent sustained elevation of IOP [40].

Although the Trendelenburg position carries risks such as increased cardiopulmonary load, intracranial pressure, intraocular pressure, and degree of gastroesophageal reflux [20, 40], no complications associated with a short duration (< 2 h) 15° Trendelenburg position have been reported [30], and no significant impact on respiratory and circulatory status under anesthesia has been observed [27]. In addition, the 15°–20° Trendelenburg position can reduce the incidence of hypotension during general anesthesia induction in patients, has a therapeutic effect on it, and can reduce the use of vasoactive drugs during general anesthesia induction [11, 30, 31]. To reduce any risks associated with the Trendelenburg position, once the central compartment surgery is completed, it is recommended to switch to the supine position for subsequent peripheral compartment surgery, minimizing the total duration of maintaining the Trendelenburg position [20].

Current limb or trunk fixation methods involved in the postless hip distraction technique mainly include upper limb fixation, contralateral lower limb fixation, and trunk fixation. The yoga mat technique [15] and the Tutankhamun technique [12] may affect anesthesia due to excessive restraint of the upper limbs and chest; in addition, long‐term fixation with the forearms crossed on the chest may cause poor venous return or infusion obstruction. This requires anesthesiologists and circulating nurses to perform regular checks, which brings certain difficulties to fluid management. Therefore, it is not recommended as the preferred method.

Relevant research reports on postless hip distraction have shown that general anesthesia is mostly used during surgery, and epidural anesthesia can also be used. General anesthesia facilitates the adjustment of anesthetic drugs and muscle relaxants, which is conducive to intraoperative operations and is preferred. Femoral nerve block is not a necessary procedure [12, 13, 14, 17].

Most studies involving hip arthroscopy have not reported the type of anesthesia (e.g., general anesthesia, nerve block, regional block, or local anesthesia) [7]. According to current literature reports on the postless hip distraction technique, general anesthesia is widely used, with or without muscle relaxants, but specific drugs have not been reported in detail. At the level of conference abstracts and expert opinions, it has been proposed and discussed that hip arthroscopy with postless hip distraction is associated with less postoperative pain [10]. However, a recent study [10] found that there is no significant difference in the amount of analgesics used before, during, and after surgery, as well as the total amount of analgesics used, between the postless hip distraction technique and the traditional post hip distraction technique. In a retrospective study by Schaver et al. [10] comparing the effects of hip traction with and without a perineal post on postoperative pain, the study included two groups, each with 100 patients, using the post hip distraction technique and the postless hip distraction technique, respectively. The study team evaluated surgical duration, analgesic drug use, pain level, and postoperative discharge time from the post‐anesthesia care unit. They found that although the pain scores and analgesic consumption were similar between the two groups, the surgical duration was significantly shorter in the postless hip distraction group (mean 11 min faster) and the postoperative discharge time from the post‐anesthesia care unit was also shorter (mean 40 min faster).

#### 3.4.6 Clinical Question 15: Is Preoperative Pre‐Traction Testing Necessary When Using the Trendelenburg Position Postless Traction Technique in Hip Arthroscopy?

*Recommendation* 18: It is recommended to perform routine preoperative pre‐traction testing. If the testing indicates that it is difficult to achieve satisfactory traction effect with the Trendelenburg position postless traction technique, the post hip distraction technique can be used instead. If significant patient displacement is observed during traction with insufficient increase in joint space, it is recommended to re‐adjust the patient's position, check whether the trunk fixation is firm, and increase the dosage of muscle relaxants as appropriate (Strong recommendation, evidence level C).

*Summary of evidence*: Preoperative pre‐traction is a necessary operation to verify the patient's position and ensure smooth surgical progress. The traction force for the hip joint without a perineal post is generally small [31, 41]; a study by Welton et al. [11] suggests that an average traction force of about 30 kg is sufficient to achieve a satisfactory effect. A distracted joint space of 8–10 mm after traction is considered sufficient for surgical manipulation [20, 26]. Previous literature reports have indicated that patients with congenital developmental hip diseases (such as excessive acetabular coverage and deep acetabular fossa) [30, 42] or inflammatory hip diseases (such as ankylosing spondylitis and severe osteoarthritis) require greater traction force during hip arthroscopy, and the risk of patient position slipping also increases accordingly. If satisfactory traction effect cannot be achieved with postless hip distraction technique, the post hip distraction technique can be used instead to avoid intraoperative accidents.

#### 3.4.7 Clinical Question 16: How to Address Insufficient Distraction of the Joint Space When Using the Trendelenburg Position Postless Hip Distraction Technique in Hip Arthroscopy?

*Recommendation* 19: Intraoperatively, if the joint space does not reach 8–10 mm, it indicates insufficient traction. In this case, the Trendelenburg angle can be moderately increased; if the joint space still cannot be sufficiently distracted, adjustments can be made according to the traditional post hip distraction method until a satisfactory traction effect is achieved (Strong recommendation, evidence level C).

*Summary of evidence*: The size of the affected hip joint space depends on factors such as the magnitude of the traction force, the Trendelenburg angle, and the dosage of muscle relaxants used. Excessively large traction force increases the risk of neuropraxia to the perineal and lateral femoral cutaneous nerves and the superficial peroneal nerve, which may lead to numbness in the medial thigh, lateral thigh, and foot, respectively [31, 32]. Therefore, a thorough preoperative physical examination and relevant auxiliary examinations, accurate assessment of the affected limb condition, and determination of an appropriate initial traction force play a crucial role in surgical outcomes and postoperative recovery. The following factors affect the magnitude of the initial traction force for the affected limb: ① Condition of the affected hip joint capsule: The degree of relaxation of the affected hip joint capsule is the most important factor affecting the traction force for the affected limb. Preoperatively, the Beighton score can be used to roughly assess the degree of relaxation of the affected hip joint capsule. If the score is lower than 2 points, a larger initial traction force for the affected limb is required [41]. In addition, an intact labrum can enhance the sealing of the hip joint capsule, thereby increasing the initial traction force for the affected limb, while conditions such as labral tears that reduce the sealing of the hip joint capsule will significantly reduce the initial distraction force. ② Perineal post use: The postless hip distraction technique can significantly reduce the traction force required to distract the joint space by eliminating the use of the perineal post and increasing the Trendelenburg angle [26].③ Gender: Male patients require a larger initial traction force for the affected limb than female patients [35]. ④ BMI: In the same gender, patients with a higher BMI require a larger initial traction force for the affected limb. ⑤ Range of motion: At the same traction force, external or internal rotation of the affected limb can increase the traction force felt by the affected hip joint. In other words, when the angles of internal rotation, external rotation, and abduction of the affected limb are small, a larger initial traction force for the affected limb is required [43].

In addition, no clear association has been found between the lateral center‐edge angle of the affected hip, developmental hip dysplasia, and the required distraction force for the affected limb [44].

#### 3.4.8 Clinical Question 17: What Are Key Perioperative Nursing Considerations for the Trendelenburg Position Postless Traction Technique in Hip Arthroscopy?

*Recommendation* 20: Nursing care for the Trendelenburg position postless hip distraction technique requires intraoperative cooperation with surgeons for patient positioning and fixation. This includes protecting bony prominences of the limbs, monitoring blood supply and skin condition of the affected limb, recording traction duration and providing timely reminders to surgeons. Postoperative attention should be paid to airway‐related risks and other conditions of patients after general anesthesia, and corresponding measures should be taken in a timely manner (Strong recommendation, evidence level C).

*Summary of evidence*: Prolonged intraoperative traction increases the risk of nerve and vascular complications. Therefore, traction duration and traction force should be recorded during surgery, and the surgeon should be reminded every half an hour. Usually, traction duration should not exceed 1.5 h, and the blood supply of the lower limbs should be observed in a timely manner. If necessary, a strategy of intermittent traction should be considered, balancing traction force and duration [36].

The ankle bears a huge traction force during traction.; therefore, when positioning the patient, the patient's feet should be wrapped with soft pads, especially near any bony prominences or potential neurovascular compression sites, to reduce irritation to the feet [45, 46]. At the same time, to avoid insufficient fixation of the feet and “slippage” during traction caused by thick cotton pads, “8”‐shaped elastic bandages can be used outside the foot after traction and fixation for reinforcement, and the toes should be left exposed to allow for observation of blood supply [47].

After surgery, nursing staff should monitor for anesthesia‐related conditions such as laryngospasm, laryngeal edema, or tongue base ptosis, and take appropriate measures in a timely manner. Then, appropriate arrangements should be made for the patient's diet, position, activities, urination, and pressure sore prevention. Ice packs can be used for local cold compresses on the surgical site to relieve pain, 30 min per time, three times a day. With the cooperation of the patient and their family members, appropriate functional exercises can be performed after the disappearance of anesthesia, and then a rehabilitation exercise plan can be formulated for the patient [43, 44, 48, 49, 50].

## Author Contributions

Jiakai Sun and Hao Fu are members of the drafting group, who participated in literature search and drafting the initial version of the manuscript. Qingfeng Yin is the corresponding author and a member of the expert panel for consensus development. Chunbao Li is the corresponding author, a member of the expert panel for consensus development, and a member of the consensus steering committee.

## Funding

The authors have nothing to report.

## Conflicts of Interest

The authors declare no conflicts of interest.

## Supporting information


**Appendix S1:** Expert Selection Protocol.

**Appendix S2:** Supplementary file 2.

## Data Availability

The data that support the findings of this study are available from the corresponding author upon reasonable request.
