# Supplementary material for: Expert Consensus on Trendelenburg Position Postless Hip Distraction Technique in Hip Arthroscopy
Source: Orthop Surg. 2026 Apr 21;18(6):1103–15. doi: 10.1111/os.70303 (PMC13238580; doi:10.1111/os.70303)
Supplement: Supplementary file 1 — Appendix S1: Expert Selection Protocol. [file OS-18-1103-s001.docx]

Expert Consensus on Postless Hip Distraction Using Trendelenburg Position in Hip Arthroscopy

Report on Expert Selection Protocol and Results

I. Expert Selection Protocol

(I) Purpose of Selection

To establish an authoritative, diverse, and conflict‑free expert panel of 40 members, ensuring the scientific rigor and clinical applicability of this consensus, guiding the clinical application of postless hip distraction techniques, and promoting advancements in hip arthroscopy.

(II) Selection Principles

Professional Appropriateness: Covering core disciplines including sports medicine, anesthesiology, and nursing, with relevant clinical and research experience.

Authoritative Qualifications: Associate senior title or above, or senior attending physician with extensive clinical experience and academic influence.

Diverse Representation: Geographically distributed across China, mainly from tertiary Class A hospitals, with balanced disciplinary coverage.

Conflict of Interest Avoidance: Signing a Conflict of Interest Statement, with no direct financial interests related to the technique.

Transparency and Traceability: Open and documented selection process; voluntary and full participation required.

(III) Selection Criteria

1.Inclusion Criteria

Engaged in relevant clinical or research fields.

Associate senior title or above, or senior attending physician with relevant academic achievements.

Geographically representative across China.

Voluntary participation and signed statements/agreements.

2.Exclusion Criteria

Direct financial conflicts of interest related to the technique.

Record of academic misconduct or professional mismatch.

Unable to guarantee full participation, with anticipated absences of ≥2 sessions.

(IV) Selection Process

A selection working group was established, and expert candidates were nominated through multiple channels.

Preliminary screening of eligible candidates was performed; the final list was determined by committee voting and publicized for 3 working days.

Invitation letters were sent to selected experts; the expert panel was formally established after relevant documents were signed.

(V) Composition of the Expert Panel

The panel consists of a Consensus Steering Committee, Consensus Development Group, Writing Expert Group, External Review Group, Corresponding Author Group, and Drafting Group, with clear division of responsibilities.

(VI) Conflict of Interest and Timeline

Conflict of interest was reviewed by the Steering Committee, with results archived and publicized.The process proceeded in sequence: nomination → preliminary review → evaluation → public notice → confirmation, to ensure efficiency.

II. Report on Expert Selection Results

(I) Basic Information

Consensus title: Expert Consensus on Postless Hip Distraction Using Trendelenburg Position in Hip Arthroscopy

Leading institution: Chinese PLA General Hospital

A 40‑member expert panel was successfully established.

(II) Selection Process and Outcomes

Strictly following the established principles and criteria, 40 candidates were unanimously approved through nomination, preliminary review, voting, and evaluation. No objections were raised during the public notice period.Experts showed balanced distribution in discipline, region, and institution; all held associate senior titles or above, with no direct conflicts of interest.

(III) List of the Expert Panel (by group, sorted by Pinyin)

1.Corresponding Authors Group:Jianquan Wang (Department of Sports Medicine, Peking University Third Hospital); Yujie Liu (Department of Sports Medicine, Orthopedic Medicine Department, Chinese PLA General Hospital); Qingfeng Yin (Department of Joint Surgery/Sports Medicine, The Second Qilu Hospital of Shandong University); Chunbao Li (Department of Sports Medicine, Orthopedic Medicine Department, Chinese PLA General Hospital).

2.Drafting Group:Yaoting Wang ,Hao Fu ,Lingxing Jiang , Jiakai Sun (Department of Sports Medicine, Orthopedic Medicine Department, Chinese PLA General Hospital).

3.Consensus Steering Committee:Liu Yujie,Li Chunbao(Department of Sports Medicine, Orthopaedic Medicine Center, Chinese PLA General Hospital)

4.Consensus Development Group:Consensus Development Expert Group (sorted by pinyin of surname): Jiangang Cao (Department of Sports Injury and Arthroscopy, Tianjin Hospital, Tianjin); Gang Chen (Sports Medicine Center, West China Hospital, Sichuan University); Guoxi Chen (Department of Orthopedics and Traumatology, Zhejiang Provincial Hospital of Traditional Chinese Medicine); Guangxing Chen (Joint Surgery Center, The First Affiliated Hospital of Army Medical University); Jiwu Chen (Department of Sports Medicine, Huashan Hospital Affiliated to Fudan University); Xingzuo Chen (Department of Traumatic Orthopedics, China-Japan Friendship Hospital); Jiangtao Dong (Department of Joint Orthopedics, The Third Hospital of Hebei Medical University); Xiaoqi Kang (Department of Sports Medicine, Orthopedic Medicine Department, Chinese PLA General Hospital); Hongyan He (Department of Sports Medicine, Orthopedic Medicine Department, Chinese PLA General Hospital); Hongjie Huang (Department of Sports Medicine, Peking University Third Hospital); Di Jia (Department of Sports Medicine, The First Affiliated Hospital of Kunming Medical University); Xiaodong Ju (Department of Sports Medicine, Peking University Third Hospital); Hongyun Li (Department of Sports Medicine, Huashan Hospital Affiliated to Fudan University); Mengmeng Li (Department of Anesthesiology, The Fourth Medical Center of Chinese PLA General Hospital); Liyun Liu (The Second Department of Hip Joint Surgery, Henan Luoyang Orthopedic-Traumatological Hospital); Yujie Liu (Department of Sports Medicine, Orthopedic Medicine Department, Chinese PLA General Hospital); Yang Liu (Sports Medicine Diagnosis and Treatment Center, Xi'an Honghui Hospital); Kan Ouyang (Department of Sports Medicine, Shenzhen Second People's Hospital); Haile Pan (The Second Department of Orthopedics, The Second Affiliated Hospital of Harbin Medical University); Decheng Shao (Department of Sports Medicine, The Third Hospital of Hebei Medical University); Geng Wang (Department of Anesthesiology, Beijing Jishuitan Hospital Affiliated to Capital Medical University); Long Wang (Department of Sports Medicine, Orthopedic Medicine Department, Chinese PLA General Hospital); Mingxing Wang (Department of Sports Medicine, Orthopedic Medicine Department, Chinese PLA General Hospital); Wenjuan Wang (Department of Sports Medicine, Orthopedic Medicine Department, Chinese PLA General Hospital); Xuesong Wang (Department of Sports Medicine, Beijing Jishuitan Hospital); Yaoting Wang (Department of Sports Medicine, Orthopedic Medicine Department, Chinese PLA General Hospital); Zhixue Wang (Decheng Shao, The Third Hospital of Hebei); Yan Xu (Department of Sports Medicine, Peking University Third Hospital); Zhihong Xu (Department of Sports Medicine and Adult Reconstruction Surgery, Nanjing Drum Tower Hospital); Denghui Xie (Department of Sports Medicine, The Third Affiliated Hospital of Southern Medical University); Zonggang Xie (Department of Orthopedics, The Second Affiliated Hospital of Soochow University); Haomiao Yu (Department of Orthopedics, Beijing Friendship Hospital Affiliated to Capital Medical University); Qingfeng Yin (Department of Joint Surgery/Sports Medicine, The Second Qilu Hospital of Shandong University); Chun Zeng (Department of Sports Medicine, The Third Affiliated Hospital of Southern Medical University); Shanxing Zhang (Department of Sports Medicine, Shanghai First People's Hospital); Xin Zhang (Department of Sports Medicine, Peking University Third Hospital); Jin Zhang (Department of Sports Injury, Beijing Jishuitan Hospital); Juanli Zhu (Department of Sports Medicine, Orthopedic Medicine Department, Chinese PLA General Hospital).

5.Writing Expert Group: Long Wang,Mingxing Wang, Yaoting Wang,Chunbao Li(Department of Sports Medicine, Orthopaedic Medicine Center, Chinese PLA General Hospital)

6.External Review Group:Yingfang Ao (Institute of Sports Medicine, Peking University Third Hospital); Shiyi Chen (Department of Sports Medicine, Huashan Hospital Affiliated to Fudan University); Chao Feng (Department of Pediatric Orthopedics, Beijing Jishuitan Hospital); Jian Li (Department of Orthopedics-Sports Medicine Center, West China Hospital, Sichuan University); Shen Liu (Department of Rehabilitation Medicine, Shanghai Sixth People's Hospital).

The selection process was standardized and transparent. The expert panel was appropriately composed and free of conflicts of interest, meeting the requirements for consensus development. Subsequent work is officially initiated.

III. Supplementary Notes

This consensus adopted the GRADE system and RIGHT guidelines. Seventeen core clinical questions were identified, and 20 evidence‑based recommendations were formulated to improve technical standardization and healthcare quality.
