# Supplementary material for: Expert Consensus on Trendelenburg Position Postless Hip Distraction Technique in Hip Arthroscopy
Source: Orthop Surg. 2026 Apr 21;18(6):1103–15. doi: 10.1111/os.70303 (PMC13238580; doi:10.1111/os.70303)
Supplement: Supplementary file 2 — Appendix S2: Supplementary file 2. [file OS-18-1103-s002.docx]

| Recommendation | GRADE Evidence Quality | Strength | Main Reason for Downgrading |
| --- | --- | --- | --- |
| 1 | C | Strong | Mainly case series and expert experience |
| 2 | D | Strong | Descriptive technical evidence |
| 3 | D | Strong | Technical definition and mechanistic reasoning |
| 4 | C | Strong | Physical and biomechanical reasoning |
| 5 | C | Strong | Non-randomized comparative cohorts |
| 6 | C | Strong | Non-randomized but highly consistent outcomes |
| 7 | C | Weak | Inconsistency across studies |
| 8 | C | Strong | Observational evidence |
| 9 | D | Weak | Expert clinical experience |
| 10 | C | Weak | Indirect economic evidence |
| 11 | C | Strong | Cohort and case-series studies |
| 12 | C | Weak | Physiological reasoning |
| 13 | C | Weak | Case reports and mechanistic analysis |
| 14 | C | Strong | Observational data |
| 15 | C | Strong | Clinical procedural experience |
| 16 | C | Strong | Standard operative protocol |
| 17 | C | Weak | Anesthetic clinical experience |
| 18 | C | Strong | High preventive value |
| 19 | C | Strong | Rescue strategy |
| 20 | C | Strong | Perioperative nursing protocol |
